# SARS-CoV-2-Specific Antibody and T Cell Response Kinetics According to Symptom Severity

**DOI:** 10.4269/ajtmh.20-1594

**Published:** 2021-06-17

**Authors:** Ji Yeun Kim, Ji-Soo Kwon, Seongman Bae, Hye Hee Cha, Joon Seo Lim, Min-Chul Kim, Jin-Won Chung, Se Yoon Park, Myung Jin Lee, Baek-Nam Kim, Jiwon Jung, Min-Jae Kim, Eui-Cheol Shin, Sung-Han Kim

**Affiliations:** 1 Department of Infectious Diseases, Asan Medical Center, University of Ulsan College of Medicine, Seoul, Republic of Korea;; 2 Graduate School of Medical Science and Engineering, Korea Advanced Institute of Science and Technology (KAIST), and Center for Epidemic Preparedness, KAIST, Daejeon, Republic of Korea;; 3 Clinical Research Center, Asan Institute for Life Sciences, Asan Medical Center, Seoul, Republic of Korea;; 4 Division of Infectious Diseases, Department of Internal Medicine, Chung-Ang University Hospital, Chung-Ang University College of Medicine, Seoul, Republic of Korea;; 5 Department of Infectious Diseases, Soonchunhyang University Seoul Hospital, Soonchunhyang University College of Medicine, Seoul, Republic of Korea;; 6 Department of Infectious Diseases, Inje University Sanggye Paik Hospital, Inje University College of Medicine, Seoul, Republic of Korea

## Abstract

Data on the longevity of humoral and cell-mediated immune responses against severe acute respiratory syndrome coronavirus 2 (SARS-CoV-2) in patients with coronavirus disease 2019 (COVID-19) are limited. We evaluated the detailed kinetics of antibody and T-cell responses at the acute, convalescent, and post-convalescent phases in COVID-19 patients with a wide range of severity. We enrolled patients with COVID-19 prospectively from four hospitals and one community treatment center between February 2020 and January 2021. symptom severity was classified as mild, moderate, or severe/critical. Patient blood samples were collected at 1 week (acute), 1 month (convalescent), and 2 months after symptom onset (post-convalescent). Human SARS-CoV-2 IgG and IgM antibodies were measured using in-house-developed ELISA. The SARS-CoV-2-specific T-cell responses against overlapping peptides of spike proteins and nucleoprotein were measured by interferon-γ enzyme-linked immunospot assays. Twenty-five COVID-19 patients were analyzed (mild, *n* = 5; moderate, *n* = 9; severe/critical, *n* = 11). IgM and IgG antibody responses peaked at 1 month after symptom onset and decreased at 2 months. IgG response levels were significantly greater in the severe/critical group compared with other groups. Interferon-γ-producing T-cell responses increased between 1 week and 1 month after symptom onset, and had a trend toward decreasing at 2 months, but did not show significant differences according to severity. Our data indicate that SARS-CoV-2-specific antibody responses were greater in those with severe symptoms and waned after reaching a peak around 1 month after symptom onset. However, SARS-CoV-2-specific T-cell responses were not significantly different according to symptom severity, and decreased slowly during the post-convalescent phase.

## INTRODUCTION

Infection with severe acute respiratory syndrome coronavirus 2 (SARS-CoV-2) causes coronavirus disease 2019 (COVID-19),^1^ which is a currently ongoing worldwide pandemic. Understanding the antibody and T-cell responses to SARS-CoV-2 in humans is crucial for characterizing the pathogenesis of COVID-19 and developing vaccines against SARS-CoV-2.

Several studies have investigated the antibody responses to SARS-CoV-2 and reported that most patients produced IgM and IgG antibodies within 15 days of symptom onset.^2–5^ IgM and IgG antibodies were developed sequentially or simultaneously,^4^ and the levels of antibodies were different according to disease severity; patients with severe symptoms had greater antibody levels than those with mild symptoms.^3,5^ However, the longevity of SARS-CoV-2-specific antibody responses are unknown. More importantly, the comparison of the longevity of SARS-CoV-2-specific T-cell responses with that of antibody responses is still lacking.

SARS-CoV-2-specific T-cell responses are essential for controlling SARS-CoV-2 through the adaptive immune response. Previous studies have examined SARS-CoV-2-specific T-cell responses,^6–13^ but they focused only on T-cell responses during the acute phase of the disease or during convalescent phase, with phenotypic characteristics and correlations of antibody responses according to disease severity. As such, there is a lack of data on the detailed kinetics of SARS-CoV-2-specific T-cell responses in terms of even short-term longevity. We thus evaluated the detailed kinetics of antibody and T-cell responses against SARS-CoV-2 at the acute (1 week from symptom onset), convalescent (1 month from the symptom onset), and post-convalescent (2 months from the symptom onset) phases in COVID-19 patients with a wide range of symptom severity, from mild to moderate to severe/critical.

## MATERIALS AND METHODS

### Patients and collection of clinical specimens.

We enrolled confirmed cases of COVID-19 prospectively who were admitted to four university-affiliated hospitals (Asan Medical Center, Chung-Ang University Hospital, Soonchunhyang University Seoul Hospital, and Inje University Sanggye Paik Hospital) in South Korea between February 2020 and January 2021. All patients agreed to peripheral blood sampling, upon which the plasma and peripheral blood mononuclear cells (PBMCs) were separated immediately and then stored in a –80°C deep freezer (plasma) or a liquid nitrogen tank (PBMCs). The blood samples were analyzed at three time points: acute phase (1 week since symptom onset), convalescent phase (1 month after symptom onset), and post-convalescent phase (2 months after symptom onset). The severity of illness was classified into three groups according to NIH classification: group 1, asymptomatic or mild; group 2, moderate; and group 3, severe or critical.^14^ This study was reviewed and approved by the ethical committee of institutional review boards of each participating institution, and all participants signed written informed consent.

### Diagnosis of COVID-19.

Diagnosis of COVID-19 was confirmed by real-time reverse transcription–polymerase chain reaction for the *RdRp*, *N*, and *E* genes of SARS-CoV-2. Viral RNA was extracted from nasopharyngeal swab specimens using the STARMag™ 96 X 4 Universal Cartridge kit (Seegene, Seoul, Republic of Korea) according to the manufacturer’s instructions. The extracted RNA was assayed with the PowerChek 2019-nCoV real-time polymerase chain reaction kit (KogeneBiotech, Seoul, Republic of Korea), which targets the *RdRp* gene of SARS-CoV-2 and the *E* gene of beta-coronavirus, or the Allplex^TM^ 2019-nCoV assay (Seegene), which targets the *RdRp* and *N* genes of SARS-CoV-2 and the *E* gene of beta-coronavirus. Cycle threshold values less than 40 for the *RdRp* gene were considered positive results.

### Measurement of IgG and IgM antibodies.

Plasma was isolated from blood specimens by centrifugation at 2,500 rpm for 10 minutes. To inactivate SARS-CoV-2, the plasma was irradiated with up to 6 million rad from a ^60^Co gamma source or treated with 0.5% Triton X-100 (Sigma-Aldrich Co., St. Louis, MO) according to the protocols described in previous studies.^15,16^

Human SARS-CoV-2 IgG and IgM antibodies were measured by an in-house-developed ELISA. Briefly, 2 μg/mL SARS-CoV-2 S1-His protein (SinoBiological, Beijing, China) was coated onto 96-well plates (MaxiSorp; Thermo Fisher Scientific, Waltham, MA) overnight at 4°C, and then the plates were blocked with 1% bovine serum albumin in phosphate-buffered saline (PBS). Plasma diluted at 1:100 and 1:1,000 for IgM, and 1:100, 1:1,000, or 1:10,000 for IgG was added and incubated for 2 hours at room temperature. Horseradish peroxidase-conjugated anti-human IgG (Jackson Immunoresearch, West Grove, PA) and IgM (MilliporeSigma, Burlington, MA) were used as secondary antibodies. The plates were developed with 3,3′,5,5′-tetramethylbenzidine substrate (Sigma-Aldrich) and the reaction was stopped with a stop solution (Sigma-Aldrich). Optical density (OD) was measured using a Spectra-Max microplate reader (Molecular Devices LCC, San Jose, CA) at 450 nm. Data are shown as relative OD values based on a 1:100 dilution factor.

To determine the cutoff values for the ELISA, we measured the mean values and SDs of the OD values from 12 negative control plasma samples that had not been exposed to SARS-CoV-2. The cutoff values were determined by calculating the mean OD plus 3-fold of the SD values, which were 0.4 for both IgG and IgM, as reported in previous studies.^17,18^

### Isolation of PBMCs.

PBMCs were isolated by density-gradient sedimentation, as described in our previous study.^19^ Briefly, peripheral blood was mixed with an equal volume of PBS and layered on top of a lymphocyte separation medium (Corning, Manassas, VA) in a 50-mL centrifuge tube. The tube was then centrifuged at 400*g* for 20 minutes at 20°C, and the lymphocyte layer was collected and washed with PBS. The PBMCs were counted, suspended in fetal bovine serum (Gibco, Grand Island, NY), and then frozen with 10% dimethyl sulfoxide (Sigma-Aldrich).

### Measurement of SARS-CoV-2-specific T-cell response.

The SARS-CoV-2-specific T-cell response was measured by interferon-γ enzyme-linked immunospot (ELISPOT) assays (T-Track human IFN-γ HiSpecificity^PRO^; Lophius Biosciences GmbH, Regensburg, Germany). The ELISPOT assays were performed with frozen PBMC samples and overlapping peptides covering the spike protein and nucleocapsid protein of SARS-CoV-2 (JPT Peptide Technologies, Berlin, Germany) dissolved in 4% dimethyl sulfoxide. PBMCs were suspended at 5.0 × 10^6^ cells/mL in RPMI1640 + 5% fetal bovine serum, and samples of 5.0 × 10^5^ cells were placed in pre-coated wells. The cells were stimulated with overlapping peptides of spike protein-1 (S1), spike protein-2 (S2), nucleocapsid protein (N), phorbol 12-myristate 13-acetate/ionomycin (Sigma-Aldrich), or medium-containing solvent, and incubated for 24 hours. The resulting spots were counted with an automated ELISPOT reader (AID *i*SPOT; Autoimmun Diagnostika GmbH, Strassberg, Germany), and the results were expressed as the number of spot-forming cells per 5.0 × 10^5^ PBMCs.

### Statistical analyses.

Categorical variables were compared using Fisher’s exact test or Pearson’s χ^2^ test as appropriate. Continuous variables were compared using the Mann-Whitney *U* test, Student’s *t*-test, or Kruskal-Wallis one-way analysis of variance with Dunn’s multiple comparison tests. The antibody levels and T-cell responses between 1 week and 1 month or 1 month and 2 months after symptom onset were compared by paired *t*-test. The clinical results were analyzed using SPSS Statistics for Windows (v. 23.0; IBM Corp., Armonk, NY). Graph plotting and analysis for the relative OD values (450 nm) and spot-forming cell data were done using GraphPad Prism 5 (GraphPad Software, San Diego, CA). All tests of significance were two tailed, and *P* values < 0.05 were considered to be significant.

## RESULTS

### Clinical characteristics of the patients.

Twenty-five patients with COVID-19 were classified according to disease severity as mild (*n* = 5), moderate (*n* = 9), and severe/critical (*n* = 11, with nine severe patients and two critical patients). Detailed baseline characteristics and clinical outcomes of the patients according to disease severity are shown in Table 1. Age and hypertension correlated highly with disease severity (*P* = 0.01 and *P* = 0.03, respectively). In the severe/critical group, one patient received convalescent plasma therapy and one patient died at the hospital.

**Table 1:** Demographic and clinical characteristics of patients with coronavirus disease 2019

Variables	Total (*n* = 25)	Mild* (*n* = 5)	Moderate† (*n* = 9)	Severe/Critical† (*n* = 11)	*P* value†
Age, y	59 (40–70)	40 (26–48)	59 (34–62)	70 (58–75)	0.01
Male gender	12 (48)	3 (60)	3 (33)	6 (55)	0.53
Comorbidity					
Diabetes	3 (12)	0 (0)	0 (0)	3 (27)	0.11
Hypertension	9 (36)	0 (0)	1 (11)	8 (73)	0.003
Cardiovascular disease	2 (8)	0 (0)	0 (0)	2 (18)	0.25
Chronic kidney disease	2 (8)	0 (0)	0 (0)	2 (18)	0.25
Chronic lung disease	2 (8)	0 (0)	0 (0)	2 (18)	0.25
Malignancy	3 (12)	1 (20)	0 (0)	2 (18)	0.38
Initial symptoms					
Fever	18 (72)	1 (20)	6 (67)	11 (100)	0.004
Chill	5 (20)	1 (20)	1 (11)	3 (27)	0.67
Cough	13 (52)	1 (20)	6 (67)	6 (55)	0.24
Sputum	4 (16)	0 (0)	1 (11)	3 (27)	0.34
Sore throat	5 (20)	1 (20)	3 (33)	1 (9)	0.40
Dyspnea	2 (8)	0 (0)	0 (0)	2 (18)	0.25
Rhinorrhea	1 (4)	0 (0)	0 (0)	1 (9)	0.52
Chest pain	1 (4)	0 (0)	1 (11)	0 (0)	0.40
Diarrhea	1 (4)	1 (20)	0 (0)	0 (0)	0.13
Headache	2 (8)	0 (0)	0 (0)	2 (18)	0.25
Myalgia	5 (20)	0 (0)	0 (0)	5 (46)	0.02
Nasal congestion	1 (4)	0 (0)	1 (11)	0 (0)	0.40
Hyposmia	6 (24)	2 (40)	4 (44)	0 (0)	0.044
Hypogeusia	6 (24)	2 (40)	4 (44)	0 (0)	0.044
Initial laboratory findings					
White blood cell count, cells/mm^3^	5,500 (4,090–6,400)	6,400 (6,380–6,600)	6,000 (5,500–6,200)	4,200 (3,520–5,250)	0.13
Hemoglobin, g/dL	13.6 (12.9–14.6)	13.2 (12.1–14.6)	13.7 (13.2–15)	13.3 (12.9–14.2)	0.71
Platelet, 10^3^/mm^3^	168 (135–223)	168 (164–234)	170 (135–234)	161 (121.5–177.5)	0.28
BUN, mg/dL	13 (10–16)	11 (9.8–13)	16 (11.8–18)	13 (10–14)	0.39
Creatinine, mg/dL	0.7 (0.6–0.9)	0.7 (0.7–0.8)	0.7 (0.6–0.9)	0.8 (0.7–0.9)	0.95
AST, IU/L	28 (24–34)	28 (21–33)	26 (24–30)	28 (25.5–43.5)	0.59
ALT, IU/L	24 (13–29)	13 (13–24)	24 (13–29)	24.0 (16.5–30.5)	0.42
CRP, mg/L	1.2 (0.7–5.5)	1.0 (0.7–1.3)	0.8 (0.3–1.3)	4.9 (1.1–5.7)	0.15
Treatment					
Lopinavir/ritonavir	6 (24)	0 (0)	1 (11)	5 (46)	0.08
Hydroxychloroquine	6 (24)	3 (60)	1 (11)	2 (18)	0.10
Remdesivir	9 (36)	0 (0)	3 (33)	6 (55)	0.11
Convalescent plasma therapy	1 (4)	0 (0)	0 (0)	1 (9)	0.52
Antibiotics	7 (28)	0 (0)	0 (0)	7 (64)	0.002
Corticosteroid	4 (16)	0 (0)	0 (0)	4 (36)	0.20
Hospital course					
Supplemental oxygen therapy	11 (44)	0 (0)	0 (0)	11 (100)	< 0.001
Mechanical ventilation	2 (8)	0 (0)	0 (0)	2 (18)	0.25
Extracorporeal membrane oxygenation	1 (4)	0 (0)	0 (0)	1 (9)	0.52
In-hospital death	1 (4)	0 (0)	0 (0)	1 (9)	0.52

BMI = body mass index; BUN = blood urea nitrogen; ALT = alanine aminotransferase; AST = aspartate aminotransferase; CRP = C-reactive protein; ICU = intensive care unit. Data are reported as *n* (%) or median (interquartile range).

* Severity was classified according to the *Coronavirus Disease 2019 (COVID-19) Treatment Guidelines* published by the NIH.

† Comparisons among groups were conducted using the χ^2^ test or the Kruskal-Wallis test.

### Kinetics of IgG and IgM antibodies.

**Figure 1.**
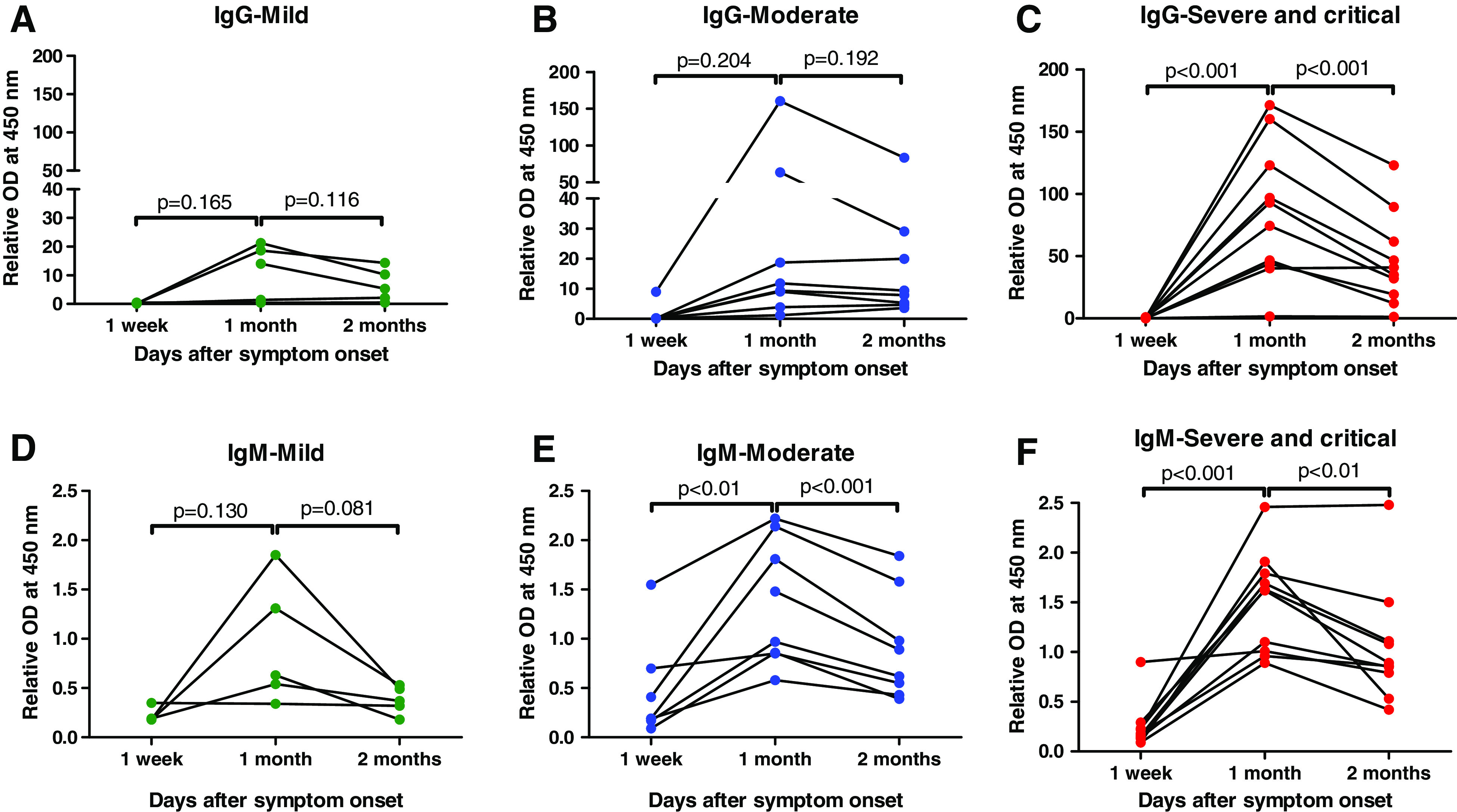
Change of IgG and IgM antibody responses against the severe acute respiratory syndrome coronavirus 2 S1 protein in paired samples classified with symptom severities. The change of IgG and IgM antibody responses in the mild group at 1 week, 1 month, and 2 months after symptom onset in (**A**, **D**) the mild group, (**B**, **E**) the moderate group, and (**C**, **F**) the severe/critical group. OD = optical density. This figure appears in color at www.ajtmh.org.

SARS-CoV-2-specific IgG and IgM antibodies were measured in 67 plasma specimens obtained from 23 patients at three time points. The IgG and IgM antibody levels showed peak levels at 1 month after symptom onset and decreased at 2 months regardless of the symptom severity (Figure 1). The IgG antibody level (mean ± SD) in the mild and moderate groups increased between 1 week (mild, 0.23 ± 0.19; moderate, 1.44 ± 3.34) and 1 month (mild, 11.15 ± 9.69; moderate, 34.73 ± 54.44) after symptom onset, then decreased at 2 months (mild, 6.49 ± 5.73; moderate, 20.42 ± 26.93), but the differences between 1 week and 1 month (mild, *P* = 0.165; moderate, *P* = 0.204), and between 1 month and 2 months (mild, *P* = 0.116; moderate, *P* = 0.192) were not significant (Figure 1A and B). Meanwhile, the IgG antibody level in the severe/critical group increased significantly between 1 week (0.24 ± 0.22) and 1 month (85.17 ± 54.76, *P* < 0.001) after symptom onset, then decreased significantly at 2 months (46.15 ± 36.96, *P* < 0.001; Figure 1C). At 1 month, the IgG response level was significantly greater in the severe/critical group compared with other groups (between mild and severe/critical, *P* < 0.01; between moderate and severe/critical, *P* < 0.05, Supplemental Figure 1A). At 2 months, the IgG response level in the severe/critical group was significantly greater than in the mild group (*P* < 0.01, Supplemental Figure 1A).

The patterns of IgM antibody levels were similar with IgG antibody levels. The IgM antibody level in the mild group increased slightly between 1 week (0.23 ± 0.08) and 1 month (0.93 ± 0.63, *P* = 0.130) after symptom onset, then decreased at 2 months (0.38 ± 0.14, *P* = 0.08), and these differences were not significant (Figure 1D). The IgM antibody levels in the moderate and severe/critical groups increased significantly between 1 week (moderate, 0.47 ± 0.52; severe/critical, 0.24 ± 0.24) and 1 month (moderate, 1.36 ± 0.64, *P* < 0.01; severe/critical, 1.51 ± 0.51, *P* < 0.001) after symptom onset, then decreased significantly at 2 months (moderate, 0.91 ± 0.54, *P* < 0.001; severe/critical, 1.05 ± 0.59, *P* < 0.01; Figure 1E and F). At 1 month, the more severe the symptoms, the greater the IgM antibody levels, but the differences between the groups were not significant. At 2 months, the IgM antibody levels in the mild group was significantly less compared with other groups (between mild and moderate, *P* < 0.05; between mild and severe/critical, *P* < 0.01; Supplemental Figure 1B).

### IFN-γ producing T-cell responses.

**Figure 2.**
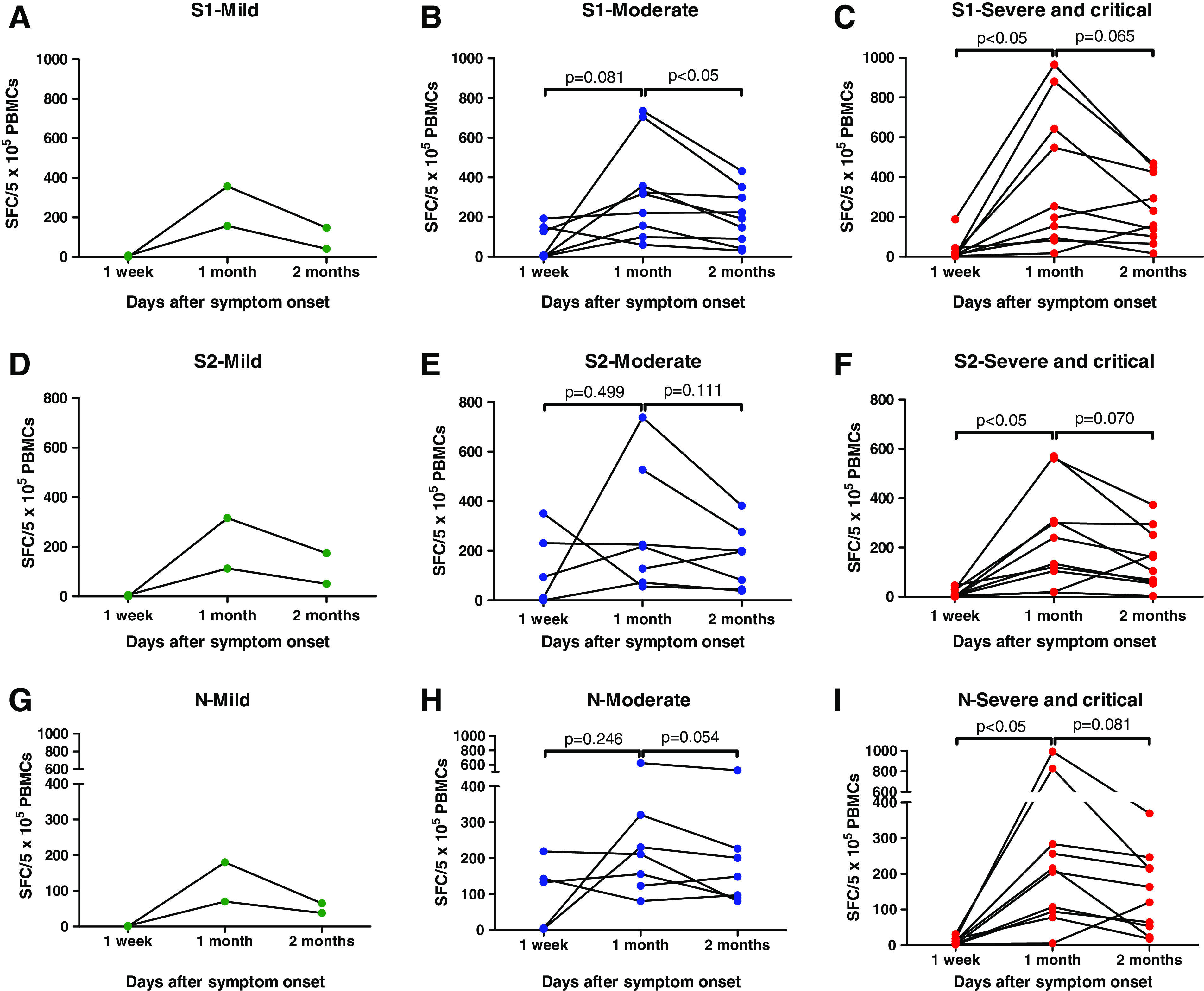
Change of interferon-γ-producing T-cell responses to severe acute respiratory syndrome coronavirus 2-specific overlapping peptides in paired samples classified with symptom severities. Spot-forming cells per 5 × 10^5^ peripheral blood mononuclear cells (PBMCs) stimulated with spike protein 1 (S1), spike protein 2 (S2), and nucleocapsid protein (N) at 1 week, 1 month, and 2 months after symptom onset in (**A**, **D**, and **G**), the mild group, (**B**, **E**, and **H**) the moderate group, and (**C**, **F**, and **I**), the severe/critical group. This figure appears in color at www.ajtmh.org.

T-cell responses were analyzed in 54 PBMC specimens obtained from 19 patients. IFN-γ-producing T-cell responses generally increased between 1 week and 1 month after symptom onset (Figure 2). In the severe/critical group, IFN-γ-producing T-cell responses increased significantly between 1 week ([mean ± SD] S1, 32.11 ± 59.95; S2, 13.78 ± 16.43; N, 11.33 ± 9.62) and 1 month (S1, 383.5 ± 348.5, *P* < 0.05; S2, 237.6 ± 200.5, *P* < 0.05; N, 306.1 ± 331.5, *P* < 0.05; Figure 2C, F, and I), but the mild and moderate groups were not significantly different between 1 week and 1 month. The IFN-γ-producing T-cell responses decreased between 1 month and 2 months after symptom onset, but the differences were not significant in all groups, except only the moderate group was stimulated with S1 (Figure 2B).

When the patients were classified according to symptom severity, IFN-γ-producing cell responses did not show significant intergroup differences at any time point after symptom onset (Supplemental Figure 2). At 1 month, IFN-γ-producing cell responses stimulated with S2 peptide in the moderate group were slightly greater than that in the severe/critical group (moderate, 280.4 ± 256.4; severe/critical, 237.6 ± 200.5; *P* = 0.718; Supplemental Figure 2).

## DISCUSSION

We investigated the SARS-CoV-2-specific humoral and cellular responses in patients with a wide range of COVID-19 symptom severity, from mild to critical, at multiple time points (i.e., acute phase, convalescent phase, post-convalescent phase). We found that the antibody levels of both IgM and IgG decreased notably between the convalescent phase and the post-convalescent phase, whereas the SARS-CoV-2-specific T-cell responses decreased slowly until the post-convalescent phase. The antibody response levels correlated closely with symptom severity, whereas SARS-CoV-2-specific T-cell responses were not significantly different according to symptom severity. Although the 2-month interval from symptom onset is somewhat short, our data provide important information during this pandemic.

Previous studies reported that the acute antibody responses to SARS-CoV-2 infection were similar to those in other viral infections, in which seroconversion takes place within 2 weeks and the IgG level peaks around 2 to 3 weeks after symptom onset.^2–5,20^ However, the kinetics of the IgM and IgG antibodies after reaching their maximum levels were yet to be clarified. Our data show that both IgM and IgG antibodies reached peak levels around 1 month after symptom onset and then decreased at 2 months after symptom onset. This change in IgG is distinct from that in SARS-CoV, which showed that the corresponding IgG levels could be maintained at high levels for up to 100 days after symptom onset.^21^

A recent study reported that SARS-CoV-2-specific antibodies decay rapidly between 30 days and 90 days after symptom onset,^22^ which is in line with our current findings. However, another study reported that antibodies against SARS-CoV-2 did not decline within 4 months after diagnosis.^23^ These discrepancies may be explained in part by the differences in the type of antibody assay and patient population, including various severities of illness. Notably, long-term follow-up studies on Middle East respiratory syndrome coronavirus and SARS-CoV showed that the corresponding antibodies persisted for 3 and 2 years, respectively.^24,25^ Therefore, further studies on the short-term and long-term antibody kinetics against SARS-CoV-2 are needed.

Several studies investigated SARS-CoV-2-specific T-cell responses in patients with COVID-19.^6–13^ Sattler et al.^11^ showed that dysfunctional T-cell responses were more common in deceased patients than in the comparators. Ni et al.^10^ reported a strong correlation between neutralizing antibody titers and SARS-CoV-2-specific T-cell responses during the convalescent period. Multiple studies showed there is some cross-reactivity of T-cell responses between SARS-CoV-2 and other beta-coronaviruses.^7,9,13^ However, there are a limited number of studies of T-cell responses based on the symptom severity of COVID-19 or the persistence of T-cell responses. Kroemer et al.^8^ reported that although the SARS-CoV-2-specific T-cell responses against the N protein were greater in those with severe illness than in those with mild illness, the T-cell responses against S protein and M protein were not significantly different according to symptom severity. Sekine et al.^12^ reported that SARS-CoV-2-specific T-cell responses in asymptomatic or mild COVID-19 patients were high in the absence of a detectable humoral response. These studies are partially in line with our findings that SARS-CoV-2-specific T-cell responses were not significantly different according to symptom severity.

It is worth noting that only a few studies investigated the longevity of SARS-CoV-2-specific T-cell responses after natural infection. One study reported the kinetics of SARS-CoV-2-specific T-cell responses during the early phase (up to 1 month after symptom onset).^13^ In our study, the SARS-CoV-2-specific T-cell responses were relatively maintained between 1 month and 2 months after symptom onset, which is somewhat different from the marked decreasing trend observed in antibody responses. These findings indicate that T-cell memory against SARS-CoV-2 may be quite durable, and that measuring T-cell immunity is a more reliable marker than antibody levels for characterizing the adaptive immune response against COVID-19. This also suggests that even if the antibody level wanes rapidly after symptom onset, the severity of illness and viral shedding upon re-exposure to SARS-CoV-2 may be minimized when T-cell immunity can be maintained.^26^ Therefore, our data provide important insight into the durable immune response against COVID-19 and vaccine development.

Our study has several limitations. First, as we did not analyze the levels of neutralizing antibodies, and it is difficult to extrapolate our data to the protective antibody response. Second, the follow-up duration (2 months after symptom onset) was somewhat short, and some patients had not been followed up until 2 months at the time of this writing. Third, we assessed the SARS-CoV-2-specific IFN-γ-releasing T-cell responses by using ELISPOT-based assays only and did not investigate the details of T-cell responses in terms of activating phenotypes, T cell subtypes, and polyfunctionality. Despite these limitations, this study is the first to evaluate the durability of humoral and cellular immune responses against SARS-CoV-2 for 2 months after symptom onset, and to analyze them according to severity of illness.

In conclusion, our data show that SARS-CoV-2-specific antibody responses were greater in those with severe symptoms and waned after reaching a peak around 1 month after symptom onset, with a decreasing trend thereafter. In contrast, SARS-CoV-2-specific T-cell responses were not significantly different according to symptom severity and were relatively maintained during the post-convalescent phase.

## References

[1] Zhu N 2020. A novel coronavirus from patients with pneumonia in China, 2019. N Engl J Med 382: 727–733. 31978945 10.1056/NEJMoa2001017 PMC7092803

[2] Sun B 2020. Kinetics of SARS-CoV-2 specific IgM and IgG responses in COVID-19 patients. Emerg Microbes Infect 9: 940–948. 32357808 10.1080/22221751.2020.1762515 PMC7273175

[3] Kara L, 2021. Magnitude and kinetics of anti-SARS-CoV-2 antibody responses and their relationship to disease severity. Clin Infect Dis 72: 301–308. 33501951 10.1093/cid/ciaa979 PMC7454426

[4] Long QX 2020. Antibody responses to SARS-CoV-2 in patients with COVID-19. Nat Med 26: 845–848. 32350462 10.1038/s41591-020-0897-1

[5] Zhao J 2020. Antibody responses to SARS-CoV-2 in patients of novel coronavirus disease 2019. Clin Infect Dis 71: 2027–2034. 32221519 10.1093/cid/ciaa344 PMC7184337

[6] Ging F 2020. Peripheral CD4+ T cell subsets and antibody response in COVID-19 convalescent individuals. J Clin Invest 130: 6588–6599. 32841212 10.1172/JCI141054 PMC7685722

[7] Grifoni A 2020. Targets of T cell responses to SARS-CoV-2 coronavirus in humans with COVID-19 disease and unexposed individuals. Cell 181: 1489–1501.e15. 32473127 10.1016/j.cell.2020.05.015 PMC7237901

[8] Kroemer M 2021. COVID-19 patients display distinct SARS-CoV-2 specific T-cell responses according to disease severity. J Infect 82: 282–327. 10.1016/j.jinf.2020.08.036 PMC7445469 32853599

[9] Le Bert N 2020. SARS-CoV-2-specific T cell immunity in cases of COVID-19 and SARS, and uninfected controls. Nature 584: 457–462. 32668444 10.1038/s41586-020-2550-z

[10] Ni L 2020. Detection of SARS-CoV-2-specific humoral and cellular immunity in COVID-19 convalescent individuals. Immunity 52: 971–977.e3. 32413330 10.1016/j.immuni.2020.04.023 PMC7196424

[11] Sattler A Angermair S Stockmann H Heim KM Khadzhynov D Treskatsch S Halleck F Kreis ME Kotsch K , 2020. SARS-CoV-2 specific T-cell responses and correlations with COVID-19 patient predisposition. J Clin Invest 130: 6477–6489. 32833687 10.1172/JCI140965 PMC7685725

[12] Sekine T 2020. Robust T cell immunity in convalescent individuals with asymptomatic or mild COVID-19. Cell 193: 158–168.e14. 10.1016/j.cell.2020.08.017 PMC7427556 32979941

[13] Weiskopf D 2020. Phenotype and kinetics of SARS-CoV-2-specific T cells in COVID-19 patients with acute respiratory distress syndrome. Sci Immunol 5: eabd2071. 32591408 10.1126/sciimmunol.abd2071 PMC7319493

[14] National Institutes of Health , 2021. *Coronavirus Disease 2019 (COVID-19) Treatment Guidelines*. Available at: https://www.covid19treatmentguidelines.nih.gov/. Accessed May 16, 2021. 34003615

[15] Hong KH 2018. Predictors of mortality in Middle East respiratory syndrome (MERS). Thorax 73: 286–289. 28724637 10.1136/thoraxjnl-2016-209313

[16] Patterson EI 2020. Methods of inactivation of SARS-CoV-2 for downstream biological assays. J Infect Dis 222: 1462–1467. 32798217 10.1093/infdis/jiaa507 PMC7529010

[17] Classen DC Morningstar JM Shanley JD , 1987. Detection of antibody to murine cytomegalovirus by enzyme-linked immunosorbent and indirect immunofluorescence assays. J Clin Microbiol 25: 600–604. 3033015 10.1128/jcm.25.4.600-604.1987 PMC266042

[18] Lardeux F Torrico G Aliaga C , 2016. Calculation of the ELISA’s cut-off based on the change-point analysis method for detection of *Trypanosoma cruzi* infection in Bolivian dogs in the absence of controls. Mem Inst Oswaldo Cruz Rio de Janeiro 111: 501–504. 10.1590/0074-02760160119 PMC4981115 27384081

[19] Kim JY 2018. Combined IFN- γ and TNF-α release assay for differentiating active tuberculosis from latent tuberculosis infection. J Infect 77: 314–320. 29746954 10.1016/j.jinf.2018.04.011

[20] Qu J Wu C Li X Zhang G Jiang Z Li X Zhu Q Liu L , 2020. Profile of immunoglobulin G and IgM antibodies against severe acute respiratory syndrome coronavirus 2 (SARS-CoV-2). Clin Infect Dis 71: 2255–2258. 32337590 10.1093/cid/ciaa489 PMC7197626

[21] Hsueh PR Huang LM Chen PJ Kao CL Yang PC , 2004. Chronological evolution of IgM, IgA, IgG and neutralisation antibodies after infection with SARS-associated coronavirus. Clin Microbiol Infect 10: 1062–1066. 15606632 10.1111/j.1469-0691.2004.01009.x PMC7129952

[22] Ibarrondo FJ Fulcher JA Goodman-Meza D Elliott J Hofmann C Hausner MA Ferbas KG Tobin NH Aldrovandi GM Yang OO , 2020. Rapid decay of anti-SARS-CoV-2 antibodies in persons with mild COVID-19. N Engl J Med 383: 1085–1087. 32706954 10.1056/NEJMc2025179 PMC7397184

[23] Gudbjartsson DF 2020. Humoral immune response to SARS-CoV-2 in Iceland. N Engl J Med 383: 1724–1734. 32871063 10.1056/NEJMoa2026116 PMC7494247

[24] Kim YS 2020. Sustained responses of neutralizing antibodies against MERS-CoV in recovered patients and their therapeutic applicability. Clin Infect Dis Sep 8: ciaa1345 (Epub ahead of print). 10.1093/cid/ciaa1345 PMC7499518 32898238

[25] Wu LP Wang NC Chang YH Tian XY Na DY Zhang LY Zheng L Lan T Wang LF Liang GD , 2007. Duration of antibody responses after severe acute respiratory syndrome. Emerg Infect Dis 13: 1562–1564. 18258008 10.3201/eid1310.070576 PMC2851497

[26] Altmann DM Boyton RJ , 2020. SARS-CoV-2 T cell immunity: specificity, function, durability, and role in protection. Sci Immunol 5: eabd6160. 32680954 10.1126/sciimmunol.abd6160

